# Thermal and Humidity Effect of Urban Green Spaces with Different Shapes: A Case Study of Shanghai, China

**DOI:** 10.3390/ijerph18115941

**Published:** 2021-06-01

**Authors:** Hongyu Du, Fengqi Zhou, Wenbo Cai, Yongli Cai, Yanqing Xu

**Affiliations:** 1 Institute of Ecology and Sustainable Development, Shanghai Academy of Social Sciences, No.7, Lane 622, Huaihaizhong Road, Huangpu District, Shanghai 200020, China; zfq@sass.org.cn; 2 Research Center for Eco-Environment Sciences, Chinese Academy of Sciences, Shuangqing Rd. 18, Beijing 100085, China; wbcai@rcees.ac.cn; 3 School of Design, Shanghai Jiao Tong University, No. 800, Dongchuan Road, Minhang District, Shanghai 200240, China; ylcai2020@sjtu.edu.cn; 4 Department of Geography & Planning, The University of Toledo, Toledo, OH 43606, USA; yanqing.xu@utoledo.edu

**Keywords:** urban green space, human thermal comfort, shape, temperature and humidity

## Abstract

Research shows that urban green spaces (UGSs) provide a number of positive effects, including enhancing human thermal comfort levels by decreasing air temperature (AT) and increasing relative humidity (RH). However, research on how the shape of an UGS influences these effects is yet to be explored. This paper explores the principles and features behind this. The AT and RH surrounding an UGS within a horizontal scale of 20 m was explored. Microclimate field measurements around 35 UGSs in Shanghai, China were carried out. The samples covered the most applied types of UGSs—punctiform, linear, and planar. Comparison spots were selected away from the sampled UGSs. The effects were studied by data collection and statistical analysis. The results indicate that the shape of the UGS had significant impact on the Temperature Humidity Index (THI). In the summer, the amplitude of THI variation decreases with the distance to UGS. For punctiform UGS, a larger total area and existence of water body results in a lower THI. A wider, linear UGS with the same orientation as the direction of the prevailing wind contributes more to decrease the surrounding THI. The total area of planar UGS is not critical. A higher landscape shape index of a planar UGS is the critical point to achieve a lower THI. The results can serve as a reference when planning and designing future UGSs.

## 1. Introduction

Urban Heat Island (UHI) aggravates the hot summer temperature and threads both the mental and physical health of citizens with extreme high temperature [1,2]. This occurs more frequently in the background of continuous urbanization around the world. Thus, increasing the human thermal comfort in summer with low energy consumption draws wide attentions. Urban Green Spaces (UGSs) play a critical role in this field by influencing the micro climate [3,4]. So, it is of great help to reveal the impact from UGS on human thermal comfort and take full advantage of the benefits.

Previous research studies have explored people’s thermal comfort perceptions in UGSs [5,6,7]. The surrounding air temperature (AT) and relative humidity (RH) of an UGS is mainly affected by evaporation, shade, and a higher ratio of radiation reflection compared to artificial impervious surfaces [8,9]. Factors affecting human thermal comfort include radiation, AT, and RH; however, UGSs can reduce UV radiation and improve AT [10].

Some of the research studies found that shading effects from green spaces, particularly trees, can increase human thermal comfort in the hot summer, and the effects are closely related to the features of the trees, such as height, canopy, diameter at breast height, and species [8,11]. Zhao, Sailor [12] evaluated the differences in outdoor microclimates and their impacts on human thermal comfort by simulating different tree layouts (clustered, equal interval, or dispersed) in the same neighborhoods. They concluded that an equal interval of two tree arrangements provided the most microclimate and human thermal comfort benefits in the neighborhood, due to the importance of shading in a hot, arid, desert environment, followed by a clustered tree arrangement (without canopy overlap). Srivanit and Jareemit [13] found (by simulation) that avenue trees can increase the outdoor thermal comfort conditions of street canyons (H/W = 0.5–0.7) by up to 82%. 

Wu, Li [14] conducted research utilizing a field measurement method, and found that the impact on temperature and humidity of green space is associated to its size. The larger the size, the lower the AT and higher RH.

Some research studies pointed out that AT and RH effects also depend on the community structure of the vegetation [11]. This shows why, in the research by Chang and Li [15], the AT and RH effects of green spaces in different types varies. In a forest area, AT and illumination is lower, but RH is higher, and has cooling and humidifying effects. In a lawn area, AT and illumination are higher, but RH is lower.

There is also research on other potential impact factors from the impervious surface ratio, the total amount of greening to the configuration of the vegetation landscape. In research by Zhang, Liu [16], using Wanshou Park in Beijing as the research area, field measurements were carried out to evaluate AT and RH features of the UGS, with pavement percentage from 0 to 40%. The results indicate that the percentage of impervious surface should be lower than 17% in order to maintain a decreasing AT and increasing RH effect of the UGS. The authors carried out another research study in Purple-bamboo Yard Park, by field measurement, indicating that, within a certain extent of green volume ratio, between an arbor, shrub, and grass, UGS with a higher quantity of green volume leads to lower AT and higher RH [17].

As for the shape of the UGS, most of the research concentrates on its impact on urban heat island mitigation. In Reference [18], the authors note that the distance of the cooling effect decreases with the LSI of the UGS. In Reference [19], the authors note that the cooling effects of UGSs with different shapes vary. However, currently, there is still a lack of research on the dependency of AT and RH improvement effects, simultaneous to the shape of green spaces. Moreover, how the UGS shapes influence these effects is of great interest. In Shanghai, the most frequently applied types of UGSs are punctiform, linear, and planar [20]. One may wonder: what are the effects of decreasing AT and increasing RH between types of UGSs? How does the shape of a planar UGS influence the AT and RH effects? These problems are yet to be solved. 

As for the research methods, currently, the most applied ones in this field are simulations [21,22,23,24], remote sensing [25], and field measurements [15]. The simulation method can eliminate the impact of multiple factors; moreover, the research concentration can be better revealed. There is plenty of research in regards to this method. De Ridder, Adamec [6], via the simulation method, carried out a research study that evaluated the benefits of green space in a well-developed city area. Evola, Costanzo [26] conducted a research study to describe a novel simulation workflow developed in the grasshopper environment, where Ladybug Tools were used to model the mutual relations amongst the urban microclimate, building energy performance, and outdoor thermal comfort. However, simulation results are usually difficult to validate, which limits the application. Moreover, simulation is generally based on the knowledge of impact principles, which makes the method not appropriate in the research of principle revealing. Remote sensing can overcome the shortage. Ige, Ajayi [1] examines the use of remote sensing and geographic information systems in mapping the Temperature Humidity Index (THI) as a human thermal comfort indicator, relative to land use/land cover change in Abuja, using the Landsat Thematic Mapper, Enhanced Thematic Mapper, and Landsat 8 Operational Land Imager/Thermal Infrared Sensor data from 1987, 1999, 2009, and 2014. However, for the remote sensing method, only surface temperature can be accurately retrieved. AT and RH cannot be acquired, which limits application of the method in human thermal comfort research. Field investigations can solve this problem. AT and RH can be accurately measured at human height and provide clear indication of thermal comfort. Galagoda, Jayasinghe [5] conducted a study that considered AT at 1 and 0.1 m distances in front of a green wall, inside the foliage, air gap, and external wall surface, comparatively to an adjacent bare wall control, via an in situ experiment. The THI modification principle of vertical green infrastructure was revealed. Thus, the field investigation method is applied in this paper.

This paper explores the relationships among UGS shapes and types with AT, RH, and human thermal comfort. A microclimate field measurement around 35 UGSs in Shanghai, China, was carried out. The effect was studied by data collection and statistical analysis. The types of UGSs covered the most applied ones—punctiform, linear, and planar. The results can serve as a reference to plan and design future UGSs.

## 2. Materials and Methods

### 2.1. Study Area

**Figure 1:**
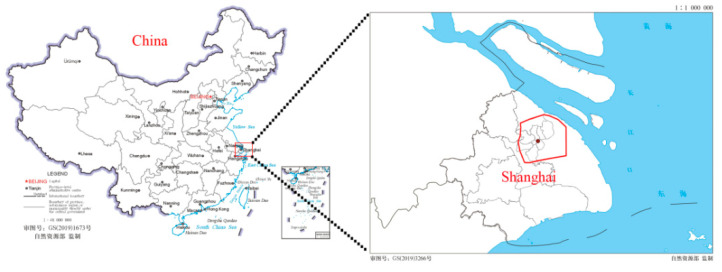
Location of study area: Shanghai, China.

Shanghai is located at Yangtze River Delta, on the east coast of China (Figure 1). It is one of the most developed and densely populated cities in China. Up until 2019, the population reached 24.2814 million. It has a humid, subtropical monsoon climate, with four distinctive seasons, including high temperatures in the summer (early June to late August). The extreme high temperatures, i.e., higher than 37 °C, appeared quite frequently in recent years. Increasing human thermal comfort in the summer season is a common concern for the public. Thus, this paper explores the THI effect of green spaces of different shapes.

### 2.2. Sample Selection

**Figure 2:**
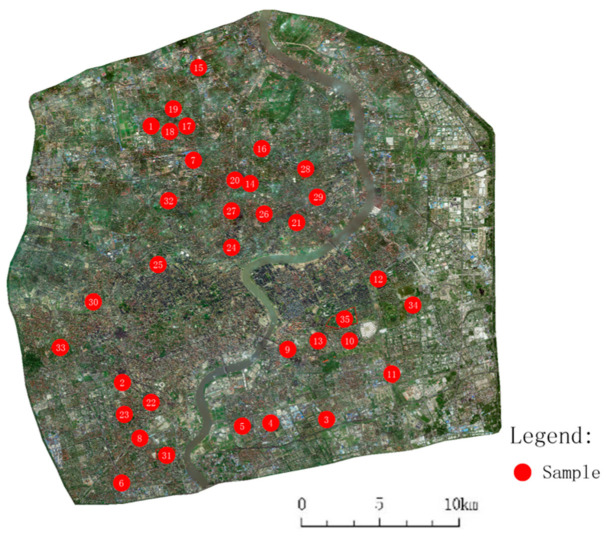
Location of the sampled UGS.

**Figure 3:**
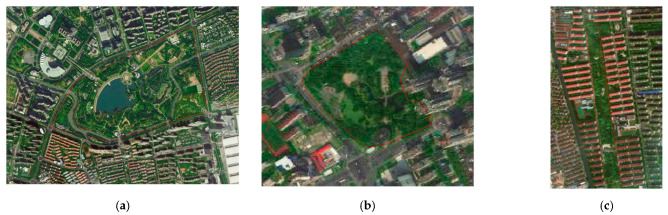
Satellite figure of UGS with different types: (**a**) planar UGS; (**b**) punctiform UGS; (**c**) linear UGS.

Three types of the most commonly applied UGSs were researched in this paper: punctiform, linear, and planar. Other UGSs that did not fall in the range of the following definition were not included in this research. Punctiform means UGS with a total area smaller than 5 ha and length–width ratio smaller than 2. Planar means UGS with a total area above 20 ha and length–width ratio smaller than 3. Linear means UGS with length–width ration higher than 5. To eliminate influences from other known impact factors, the selected samples of each type had similar greening rates and surrounding environments. Finally, the selected samples included 10 punctiform UGSs, 15 linear UGSs, and 10 planar UGSs. Details of the sampled UGSs are listed in Table 1. The distribution of the sampled UGSs are shown in Figure 2. Figure 2 presents the study area within the outer ring road (solid red line in Figure) of Shanghai. Typical satellite figures of each type of UGS are shown in Figure 3.

### 2.3. Measurement

**Figure 4:**
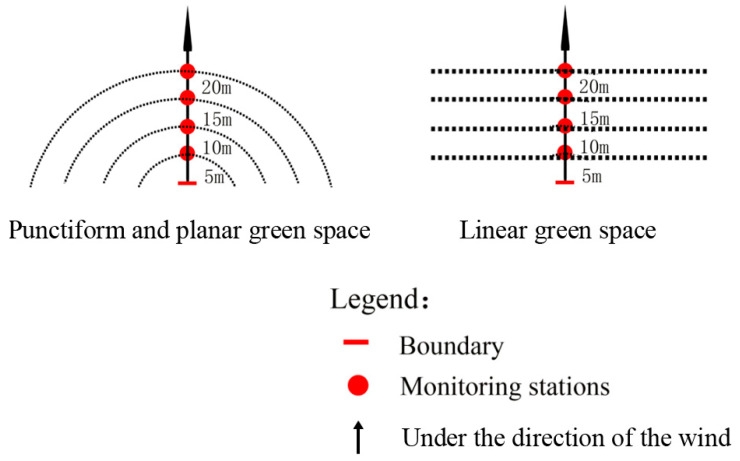
Ribbon buffers with width of 5 m in the research.

To evaluate the AT and RH of the surrounding environment, a ribbon buffer with a width of 5 m was sliced, as shown in Figure 4. All measurements were acquired from the downwind side. The TES-1365 humidity–temperature meters were distributed in each buffer at a height of 1.5 m. Meanwhile, another set of TES-1365 were mounted at reference points. Reference points were selected from typical artificial impervious surfaces, which were 80 m away from the selected UGSs. The precision of the TES-1365 m was carefully calibrated before the measurement to ensure reliable results. Each UGS, limited to the labor resources, was only measured within one day. However, the weather differences can be eliminated, as usually, in Shanghai, hot summer weather is very stable for several days. The field measurements proceeded in the daytime (9:00 a.m.–6:00 p.m.) between 27 July and 30 August 2020, when the weather was clear, dry, and calm (i.e., weak or no wind). Measurement frequency of TES-1365 was set to 30 min. All data were then collected for follow-up analysis.

### 2.4. Data Processing

After data collection (from the equipment), the Temperature Humidity Index (THI) was calculated according to Equation (1). In the equation, T is the air temperature (°C), RH is the relative humidity (%). A comparison table of THI and human thermal comfort is shown in Table 2 [27]. In order to indicate the shape of the UGS, the Landscape Shape Index (LSI) of the planar UGS was calculated using FRAGSTATS 4.2 software. LSI is defined as the ratio between the perimeter and area (Equation (2)). P (m) is the perimeter, S (m^2^) is the total area. SPSS v21 is applied in this research for statistical analysis. Two types of correlation analyses are applied in this research. The Pearson method is used to handle continuous variables while Spearman is used to handle scattered variables.

THI = T − 0.55 (1 − 0.01RH) (T − 14.5)(1)

LSI = P/S(2)

## 3. Results

### 3.1. Linear Green Spaces

**Figure 5:**
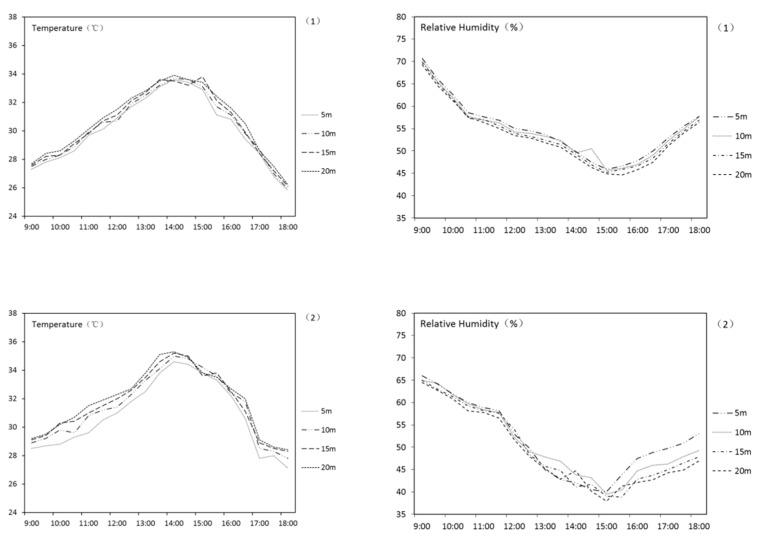

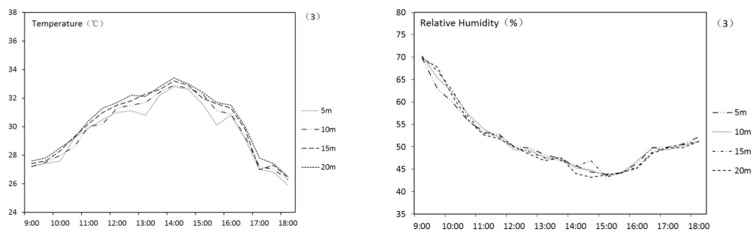
AT and RH curve of UGS nos. 1–3.

The results of the linear UGS are shown in Table 3. According to the table, linear UGS has an effect of lowering temperature and increasing humidity. On average, the linear UGS in this research can lower the surrounding AT by 2.1 °C and increase RH by 1.8%. Figure 5 shows the AT and RH curve of UGS numbers 1–3. It can be indicated that, in general, the effect fades along with the increase of the distance to UGS. A correlation analysis was carried out to reveal the relationship among the width, the orientation of linear UGS, and the THI variation. The result is shown in Table 4. This shows that the final THI variation effect in the summer from linear UGS negatively correlates to the UGS width and the orientation.

### 3.2. Punctiform Green Spaces

**Figure 6:**
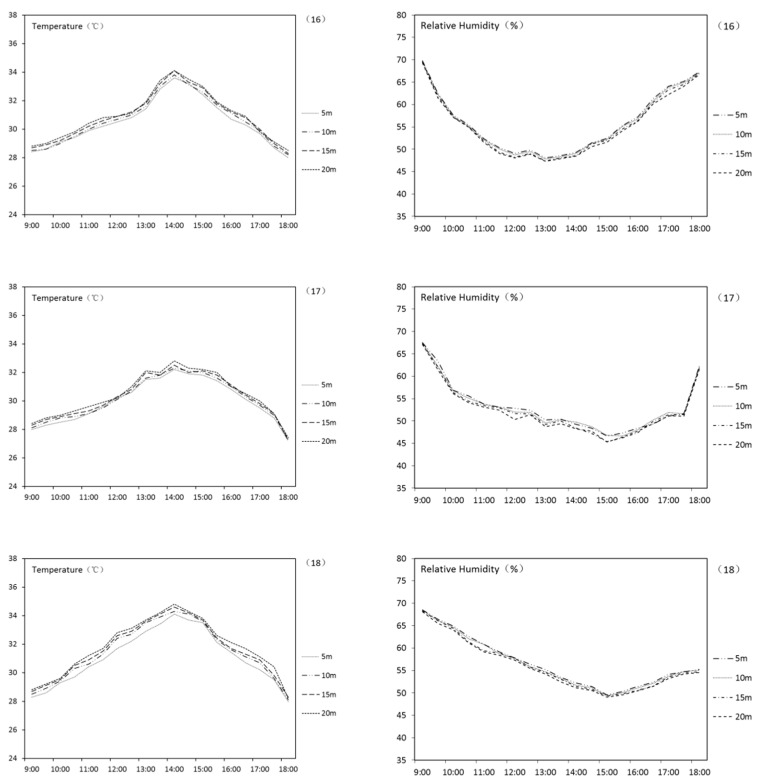
AT and RH curve of UGS nos. 16–18.

The results of THI variation of punctiform UGS are shown in Table 5. According to the table, all punctiform UGSs have an obvious effect of decreasing THI in the summer. The result is formed by simultaneous decreasing of AT and increasing of RH. Thus, human thermal comfort is increased. On average, the punctiform UGS in this research can lower the surrounding AT by 2.8 °C and increase RH by 3.0%. Figure 6 shows the AT and RH curve of UGS numbers 16–18. It can be indicated that the effect generally fades along with the increase of the distance to UGS. In particular, if there are water bodies in the punctiform UGSs, the THI improvement effect will be even better. A correlation analysis was carried out to reveal the relationship among total area, existence of the water body, LSI, and the final THI variation of UGS. Results are shown in Table 6. This indicates that the variation of THI negatively correlated to the existence of the water body and positively correlated to the total area. The influence from LSI is not as significant.

### 3.3. Planar Green Spaces

**Figure 7:**
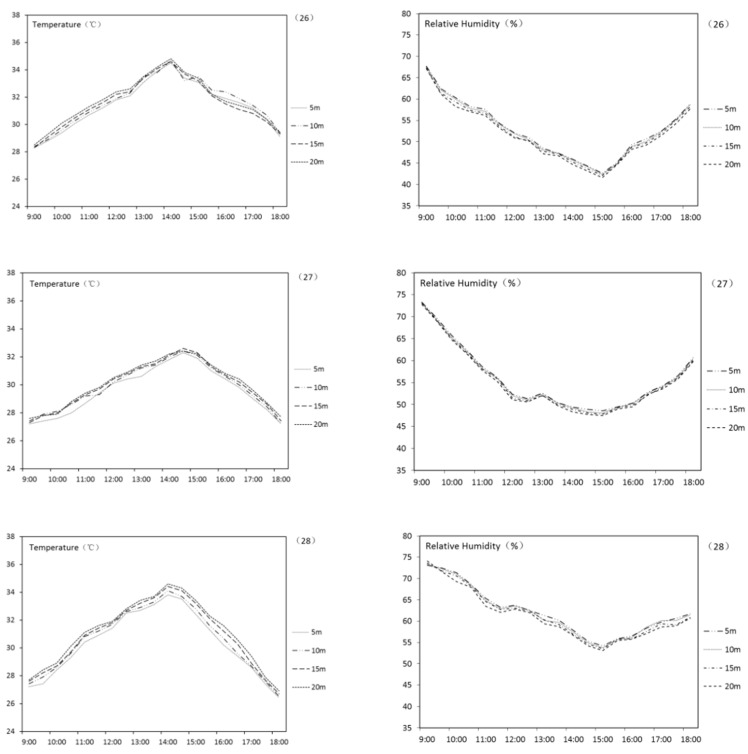
AT and RH curve of UGS nos. 26–28.

The results of planar green spaces are shown in Table 7. According to the table, planar UGS has an obvious effect of lowering AT and increasing RH. On average, the planar UGS in this research can lower the surrounding AT by 3.1 °C and increase RH by 3.8%. Figure 7 shows the AT and RH curve of UGS numbers 26–28. It can be indicated that the effect fades along with the increase of the distance to UGS. A correlation analysis was carried out to reveal the relationship among the total area, the existence of the water body, LSI, and the final THI variation of UGS. Results are shown in Table 8. This indicates that the THI variation of planar UGS in the summer positively correlated to LSI, which means an UGS with a complicated shape. The impact from total area of the UGS is not that significant.

## 4. Discussion

In general, all types of UGSs in this research had an effect with THI increase. This complies with the research results conducted in [5]. Among the three types of UGS, planar UGS has a better overall THI decrease in the summer, followed by punctiform UGS. Linear UGS has the lowest effect of THI decrease. 

For punctiform UGS, the main impact factor of THI variation was the total area, corresponding to other research that mentioned the THI decrease effect was positively impacted by the total area [14]. In addition, if a water body exits in the UGS, the THI decrease effect significantly enhances. The water body can contribute vapor to influence the RH [28]. Meanwhile, the cooling effect of the water body cools down the surrounding environment [29]. LSI is not as impactful to the AT and RH of punctiform UGS. Compared to planar UGS, punctiform UGS has a smaller size. The shape of UGS in such a small scale cannot change the general conditions of heat and water exchange. Thus, LSI is not significant in regards to the effects. Planar UGSs in this research did not show obvious THI decrease variations with differences of total areas. Specifically, the shape of planar UGS had more of an impacting factor than total area. A larger contact area with a surrounding environment enhanced the energy exchange, leading to the result. Similar to the results by Zhu, Ji [2], wider linear UGSs bring lower THI in the summer. Moreover, it was found that orientation of linear green space also had a significant impact on the THI variation. This was due to the climate feature of Shanghai. Prevailing wind are southeastern in the summer. The linear green space with the same orientation can form the corridors and increase the heat and vapor exchange [3]. Thus, the THI is lower.

## 5. Limitation and Future Work

The research was conducted by field measurement and data analysis of 35 UGS samples in Shanghai, China. Although results and conclusions are clear, the research is far from perfect. Due to the limitations, future work is necessary.

First, limited to the research time and labor resources, the number of UGS samples was few. Although the statistical analysis was not quite influenced by this, more samples in the future will improve the robustness of the conclusions.

Second, increasing the distance to UGSs will decrease the cooling effects of UGSs, but other factors could contribute to the variation of AT and RH. Efforts were made to eliminate such impacts by choosing UGSs with similar greening rates, weather conditions, and surrounding conditions. However, this was still general and not accurate enough compared to the complexity of the research topic. Finding all factors that influence the AT and RH effects of UGSs is a huge project that will require more resources and energy in the future.

Finally, in exception for the field measurement, other methods in the future should be introduced to proceed with further research. Comparisons among the different methods can provide “proof” for each one and strengthen the conclusions.

## 6. Conclusions

This research aimed to reveal how the shapes of punctiform, planar, and linear UGSs influence the THI of the surrounding environment. The method used was field microclimate measurement. The major conclusions are as follows:

The shape of the UGS has a significant impact on the surrounding THI. The amplitude of THI variation decreases with the distance to the UGS. For punctiform UGS, a larger total area and existence of water body results in a lower THI. A wider linear UGS, with the same orientation as the direction of the prevailing wind, contributes more to decrease the surrounding THI in the summer. The total area of the planar UGS is not critical. Higher LSI of a planar UGS is critical to achieve a lower THI in the summer.

It is recommended to the urban planning or greening bureau that punctiform UGS be designed with a reasonable area. If possible, a water body should be included. The orientation of the UGS should be similar to the direction of the prevailing wind (except when increasing the width of the linear UGS). Planar UGS should be designed actively with higher LSI.

## Figures and Tables

**Table 1:** List of the UGS samples.

No.	Type	Area (m^2^)	LSI	Existence of Water Body?	Orientation	Width (m)
1	Linear	56,581	1.6667	No	NE–SW	121
2	Linear	59,542	1.5	No	N–S	86
3	Linear	35,951	1.625	No	E–W	57
4	Linear	82,552	1.75	No	N–S	97
5	Linear	23,790	1.8	No	E–W	50
6	Linear	71,997	1.2682	No	N–S	89
7	Linear	21,390	1.4545	No	E–W	61
8	Linear	80,505	1.75	No	NE–SW	150
9	Linear	86,290	1.4531	No	NW–SE	105
10	Linear	41,827	1.875	No	NW–SE	124
11	Linear	35,633	1.6667	No	NW–SE	100
12	Linear	79,394	1.4074	Yes	E–W	137
13	Linear	13,124	1.15	No	E–W	35
14	Linear	20,467	1.5455	No	NE–SW	28
15	Linear	180,186	1.7895	No	N–S	178
16	Punctiform	45,328	1.6479	Yes	-	-
17	Punctiform	38,580	1.5477	Yes	-	-
18	Punctiform	24,920	1.222	No	-	-
19	Punctiform	30,843	1.4658	No	-	-
20	Punctiform	49,220	1.5117	Yes	-	-
21	Punctiform	37,807	1.3728	No	-	-
22	Punctiform	41,788	1.5923	Yes	-	-
23	Punctiform	36,595	1.2825	No	-	-
24	Punctiform	41,481	1.9987	No	-	-
25	Punctiform	37,871	1.361	No	-	-
26	Planar	203,284	1.3419	Yes	-	-
27	Planar	216,501	2.5619	Yes	-	-
28	Planar	317,904	1.1064	Yes	-	-
29	Planar	481,785	2.3488	Yes	-	-
30	Planar	465,235	1.941	Yes	-	-
31	Planar	1,336,123	2.3369	Yes	-	-
32	Planar	383,346	1.449	Yes	-	-
33	Planar	397,273	1.6112	Yes	-	-
34	Planar	200,155	2.4589	Yes	-	-
35	Planar	378,318	2.0282	Yes	-	-

**Table 2:** Comparison table of THI and human thermal comfort.

THI	Human Thermal Comfort	Evaluation Criteria
>30	Extreme hot	Cannot work without extra cooling
26.5 < THI < 30	Very hot	Very uncomfortable
20.0 < THI < 26.5	hot	Uncomfortable
15 < THI < 20	Mild	Comfort

**Table 3:** THI variation of linear UGS.

No.	Width (m)	Orientation	AT (°C)	RH (%)	Ref THI	Buffer THI				THI Variation			
						5 m	10 m	15 m	20 m	5 m	10 m	15 m	20 m
1	121	NE–SW	33.9	54.9	29.09	26.59	26.73	27.07	27.17	−2.5	−2.36	−2.02	−1.92
2	86	N–S	34.4	48.2	28.73	26.29	26.59	26.84	26.94	2−.44	−2.14	−1.89	−1.79
3	57	E–W	33.6	48.6	28.20	25.89	25.97	26.16	26.36	−2.31	−2.23	−2.04	−1.84
4	97	N–S	34.6	47.9	28.84	26.04	26.25	26.6	26.92	−2.8	−2.59	−2.24	−1.92
5	50	E–W	35.3	51.0	29.69	27.69	27.94	28.25	28.6	−2	−1.75	−1.44	−1.09
6	89	N–S	33.6	46.5	27.98	25.52	25.81	26.31	26.98	−2.46	−2.17	−1.67	−1
7	61	E–W	35.1	51.3	29.58	27.17	27.48	27.93	28.57	−2.41	−2.1	−1.65	−1.01
8	150	NE–SW	33.4	48.4	28.04	25.01	25.48	26.06	26.63	−3.03	−2.56	−1.98	−1.41
9	105	NW–SE	33.6	48.3	28.17	24.67	25.18	25.76	26.29	−3.5	−2.99	−2.41	−1.88
10	124	NW–SE	33.1	48.5	27.83	23.5	24.2	25.12	25.76	−4.33	−3.63	−2.71	−2.07
11	100	NW–SE	33.7	50.8	28.50	24.73	25.54	26.25	27.12	−3.77	−2.96	−2.25	−1.38
12	137	E–W	33.7	46.2	28.02	24.52	25.31	26.07	26.78	−3.5	−2.71	−1.95	−1.24
13	35	E–W	34.6	48.5	28.91	26.96	27.3	27.61	27.98	−1.95	−1.61	−1.3	−0.93
14	28	NE–SW	32.8	55.3	28.30	27.65	27.9	27.98	28.04	−0.65	−0.4	−0.32	−0.26
15	178	N–S	34.2	54.7	29.29	25.49	26.2	27.15	27.45	−3.8	−3.09	−2.14	−1.84

The summer prevailing wind orientation in Shanghai is NW–SE > E–W> N–S > NE–SW. Thus, the orientation of UGS is coded as 0 = NE–SW, 1 = N–S, 2 = E–W, 3 = NW–SE. Bigger variables mean closer to the summer wind orientation.

**Table 4:** Correlation analysis of linear UGS THI variation and the impact factors.

		Orientation	Width
THI variation	Correlation	−0.515 *	−0.788 **
	Significance (double direction)	0.049	0.001
	N	15	15

* Indicates significant at a level of 0.05 (double direction). ** Indicates significant at a level of 0.01 (double direction).

**Table 5:** THI variation of punctiform UGS.

No.	Area (m^2^)	LSI	WB	AT (°C)	RH (%)	Ref THI	Buffer THI				THI Variation			
							5 m	10 m	15 m	20 m	5 m	10 m	15 m	20 m
16	45,328	1.6479	Yes	34.5	53.6	29.40	26.58	26.67	26.79	26.84	−2.81	−2.72	−2.60	−2.56
17	38,580	1.5477	Yes	32.6	52.4	27.86	25.97	26.37	26.44	26.49	−1.89	−1.50	−1.43	−1.37
18	24,920	1.222	No	32.7	53.4	28.04	27.09	27.32	27.43	27.54	−0.94	−0.72	−0.61	−0.5
19	30,843	1.4658	No	33.1	50.2	28.01	26.97	27.13	27.27	27.41	−1.04	−0.88	−0.74	−0.6
20	49,220	1.5117	Yes	34.5	53.6	29.40	26.58	26.67	26.79	26.84	−2.81	−2.72	−2.60	−2.56
21	37,807	1.3728	No	33.7	50.8	28.50	26.66	26.75	26.87	27.05	−1.85	−1.75	−1.63	−1.45
22	41,788	1.5923	Yes	34.1	52.3	28.96	26.77	26.92	27.06	27.09	−2.19	−2.04	−1.90	−1.87
23	36,595	1.2825	No	33.6	51.9	28.55	26.58	26.66	26.88	27.13	−1.96	−1.88	−1.67	−1.42
24	41,481	1.9987	No	34.3	52.2	29.09	26.91	27.08	27.25	27.41	−2.18	−2.02	−1.85	−1.68
25	37,871	1.361	No	33.3	52.1	28.35	26.43	26.50	26.67	26.94	1.91	1.85	1.68	−1.41

Water body is coded as 1 = yes, 0 = no.

**Table 6:** Correlation analysis of punctiform UGS THI variation and the impact factors (5 m parameters are used).

		Area	WB	LSI
THI variation	Correlation	0.970 **	−0.649 *	−0.485
	Significance (double direction)	0.000	0.042	0.156
	N	10	10	10

* Indicates significant at a level of 0.05 (double direction). ** Indicates significant at a level of 0.01 (double direction).

**Table 7:** THI variation of planar UGS.

No.	Area (m^2^)	LSI	AT (°C)	RH (%)	Ref THI	Buffer THI				THI Variation			
						5 m	10 m	15 m	20 m	5 m	10 m	15 m	20 m
26	203,284	1.3419	34.1	50.2	28.73	25.42	26.08	26.61	27.46	−3.31	−2.65	−2.12	−1.27
27	216,501	2.5619	32.8	51.0	27.87	23.46	24.34	25.05	26.17	−4.41	−3.53	−2.82	−1.69
28	317,904	1.1064	33.1	53.9	28.38	26.23	26.66	27.01	27.56	−2.15	−1.72	−1.38	−0.83
29	481,785	2.3488	32.7	54.9	28.19	22.84	23.91	24.76	26.13	−5.35	−4.28	−3.42	−2.05
30	465,235	1.941	32.7	54.3	28.13	25.15	25.74	26.22	26.98	−2.98	−2.38	−1.91	−1.14
31	1,336,123	2.3369	33.4	55.3	28.75	23.98	24.94	25.70	26.92	−4.77	−3.82	−3.05	−1.83
32	383,346	1.449	34.3	50.8	28.94	24.44	25.34	26.06	27.21	−4.50	−3.60	−2.88	−1.73
33	397,273	1.6112	32.9	51.2	27.96	24.31	25.04	25.63	26.56	−3.65	−2.92	−2.34	−1.40
34	200,155	2.4589	33.5	50.5	28.33	23.02	24.08	24.93	26.29	−5.31	−4.25	−3.40	−2.04
35	378,318	2.0282	33.6	53.5	28.72	25.71	26.31	26.79	27.56	−3.01	−2.41	−1.93	−1.16

**Table 8:** Correlation analysis of planar UGS THI variation and the impact factors.

		Area	LSI
THI variation	Correlation	−0.232	−0.701 *
	Significance (double direction)	0.519	0.024
	N	10	10

* Indicates significant at a level of 0.05 (double direction).
